# The Use of Triaxial Accelerometers and Machine Learning Algorithms for Behavioural Identification in Domestic Dogs (*Canis familiaris*): A Validation Study

**DOI:** 10.3390/s24185955

**Published:** 2024-09-13

**Authors:** Cushla Redmond, Michelle Smit, Ina Draganova, Rene Corner-Thomas, David Thomas, Christopher Andrews

**Affiliations:** School of Agriculture and Environment, Massey University, Palmerston North 4410, New Zealand; cushla-r-@hotmail.com (C.R.); m.smit@massey.ac.nz (M.S.); i.draganova@massey.ac.nz (I.D.); r.corner@massey.ac.nz (R.C.-T.); c.j.andrews@massey.ac.nz (C.A.)

**Keywords:** algorithm, behaviour classification, overall activity, random forest

## Abstract

Assessing the behaviour and physical attributes of domesticated dogs is critical for predicting the suitability of animals for companionship or specific roles such as hunting, military or service. Common methods of behavioural assessment can be time consuming, labour-intensive, and subject to bias, making large-scale and rapid implementation challenging. Objective, practical and time effective behaviour measures may be facilitated by remote and automated devices such as accelerometers. This study, therefore, aimed to validate the ActiGraph^®^ accelerometer as a tool for behavioural classification. This study used a machine learning method that identified nine dog behaviours with an overall accuracy of 74% (range for each behaviour was 54 to 93%). In addition, overall body dynamic acceleration was found to be correlated with the amount of time spent exhibiting active behaviours (barking, locomotion, scratching, sniffing, and standing; R^2^ = 0.91, *p* < 0.001). Machine learning was an effective method to build a model to classify behaviours such as barking, defecating, drinking, eating, locomotion, resting-asleep, resting-alert, sniffing, and standing with high overall accuracy whilst maintaining a large behavioural repertoire.

## 1. Introduction

Domestic dogs (*Canis familiaris*) have become an integral part of human society, serving various purposes such as companionship, working, hunting, research, and military service roles [1,2]. Assessing their behaviour is critical for predicting suitability and targeting specific temperament characteristics required for these specific roles [3]. Importantly, behavioural assessments not only facilitate selection of desirable traits, but also enhance our understanding of the overall welfare state of dogs [4,5,6]. Common methods of behavioural assessment include test batteries, observational methods, and questionnaires [7,8,9]. These methods, however, can be time consuming, labour intensive, and subject to bias, making large-scale implementation challenging [10,11,12,13,14,15].

There is a need for methods of behavioural assessment that are objective, practical and do not require extensive time or training from human observers [16]. Recent technological advances have facilitated remote and automated behavioural measurement. Pedometers were among the earliest technologies used to measure physical activity objectively [17]. Although relatively inexpensive and easy to use, pedometers cannot measure intensity or differentiate movement patterns due to their simple design [17,18]. On the other hand, accelerometer devices offer a far more detailed behavioural assessment by recording the intensity, frequency, and duration of every movement across three axes: X, Y, and Z [13,19,20].

Recent advances in accelerometer technology have seen a reduced device size, enabling their use in monitoring the activity of companion animals [21,22]. Numerous studies have demonstrated that accelerometers can provide objective and accurate measurements of activity levels in companion animals [14,19,23,24,25,26,27,28]; however, fewer have determined specific behaviours [21,23,27,28]. ActiGraph devices have been previously validated in dogs and are tri-axial accelerometers, allowing for omnidirectional movement to be detected [25,27,28]. This allows the detection of both dynamic and static behaviours, providing a comprehensive view of the dog’s overall activity.

The application of accelerometers can enable objective quantification of various aspects of dog behaviour, including overall daily activity, resting periods, changes in behaviour, postural changes, and even movement patterns associated with each behaviour using machine learning analytical methods [29,30]. Machine learning (ML) allows for complex data sets, such as those produced by accelerometers, to be analysed using complementary data modelling techniques [31]. ML models to identify dog behaviours have been reported to have accuracies of between 69% and 97% [27,28,29,32,33]. Currently for behaviour ML models there is no accepted threshold at which a model is deemed to have reached an acceptable level of accuracy. A comparison of machine learning methods for human and animal derived accelerometer data reported that random forest models had higher accuracy than artificial neural network, k-nearest neighbours, linear discriminant analysis, naïve Bayes and support vector machine [34,35].

On a wider scale, modern technology such as accelerometers also have the potential to be used for identification of stress related behaviours, gait analysis, and health monitoring [16,22,30,36,37,38,39,40,41,42]. Therefore, this study aimed to validate the ActiGraph^®^ accelerometer as a tool of behavioural classification for domestic dogs.

## 2. Materials and Methods

This study was conducted at Massey University Canine Nutrition Unit (CNU), Palmerston North, New Zealand (latitude 40°230′ S, longitude 175°365′ E) from August to September 2023. All research was conducted in accordance with Massey University Animal Ethics Committee (MUAEC) protocol number 23/27. All husbandry of the dogs complied with MUAEC protocol number 21/25 and the Animal Welfare Code of Welfare: Dogs [43].

### 2.1. Animal Husbandry

Six healthy and desexed domesticated dogs (two female and four male) were used for this study (Table 1). The dogs aged from 6.02 ± 1.59 years (mean ± SD). The dogs participating in the study were all healthy with body condition scores from five to six (out of a nine-point scale; [44]) and bodyweights of 25.98 ± 4.27 kg (mean ± SD). Dogs were housed in specific pairs (based on behavioural compatibility) prior to and throughout the study (Table 1). During the day (07:00 h to 16:00 h), the pairs of dogs were placed in outdoor exercise paddocks that measured 12.0 × 9.5 m. Overnight (16:00 h to 07:00 h), the pairs of dogs were housed in centrally heated indoor runs.

The dogs were fed a complete and balanced adult maintenance diet (Black Hawk Working Dog, Masterpet Corporation Ltd., Lower Hutt, New Zealand). Feed quantity was determined by the dog’s maintenance energy requirements and body condition score. Normally, the dogs were fed once daily. In the present study, however, the diet was fed across three meals throughout the day, twice in the morning (between 09:00 a.m. and 10:00 a.m.) and once in the afternoon (between 15:00 p.m. and 16:00 p.m.) to increase the frequency of feeding behaviours. The dogs had *ad libitum* access to water throughout the study.

### 2.2. Experimental Design

**Figure 1:**
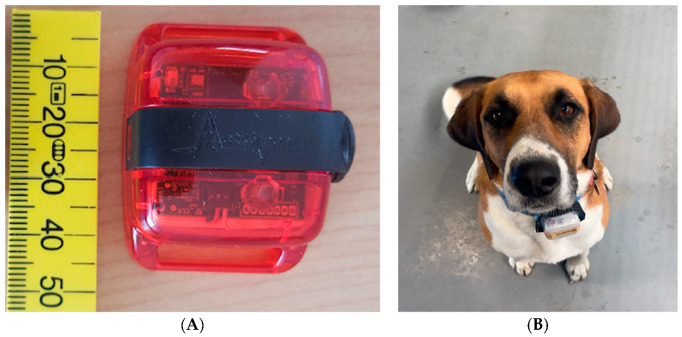
(**A**) ActiGraph^®^ wGT3X-BT device (**B**) Ventral view of the ActiGraph^®^ wGT3X-BT accelerometer, which was consistently orientated, placed within a protective housing, and fitted ventrally to the collars of the dogs.

**Figure 2:**
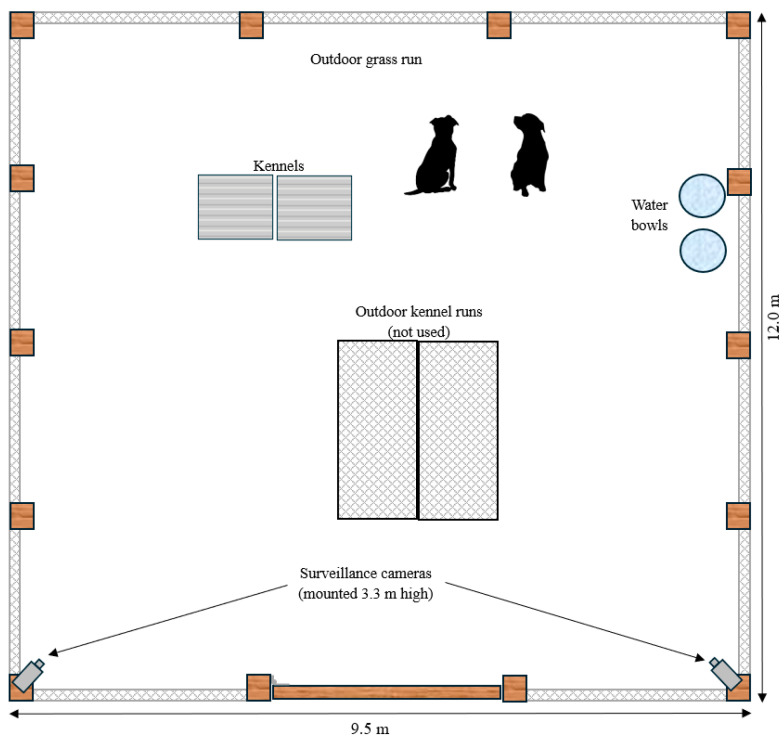
Diagram of the outdoor observation paddock showing its dimensions and features, including the positioning of the two surveillance cameras used to monitor the animals. Note that the image has not been drawn to scale.

For each pair of dogs, there was a one-day habituation phase followed by a three-day data collection phase. This process was repeated sequentially for each pair of dogs. During the habituation phase, ActiGraph^®^ wGT3X-BT accelerometers (ActiGraph, Pensacola, FL, USA) weighing 19 g and measuring 33 mm x 46 mm x 15 mm were placed inside a protective casing. These types of accelerometer devices have been extensively used in animal studies (see reviews [45,46]). The casing was then attached to their existing collar and positioned ventrally (Figure 1). The orientation of the accelerometers was uniform for all animals with the plastic lock screw positioned caudally (Figure 1). The dogs were then moved into the outdoor observation paddock (Figure 2) and monitored to ensure there were no noticeable adverse effects of the device attachment on their behaviour or well-being such as trying to scratch or bite the device. At the end of the habituation day, the collars were removed from the dogs overnight and refitted the following morning for the start of the data collection phase.

During the data collection phase, ActiGraph^®^ accelerometers were fitted to the collars of each pair of dogs and continuous triaxial acceleration data were collected at 30 Hz (see Section 2.3). The dogs were then placed in the outdoor observation pen (Figure 2). The observation paddock was under continuous video surveillance (see Section 2.4) during daylight hours for a total of eight hours per day and each pair of dogs being observed for a total of three days. This process was repeated sequentially for each subsequent pair of dogs. Thus, a total of 24 h of concurrent video footage and acceleration data were collected from each dog, totalling 144 h of data from all six animals.

### 2.3. Accelerometry (ActiGraph^®^ wGT3X-BT)

The ActiGraph^®^ accelerometers were attached to the existing collars of the dogs and used to assess the movement of dogs. To protect the devices, they were encased in a plastic container and filled with bubble wrap to protect them from damage and to avoid movement of the devices within the plastic casing (Figure 2). Waterproof tape was wrapped around the ActiGraph^®^ devices to protect them from water damage. Collar tightness was kept as consistent as possible among the dogs to reduce residual movement of the devices. The total weight of the collar and device inside the plastic casing was 115.2 g, which equated to ~0.4% of the dog’s bodyweight.

The ActiGraph^®^ wGT3X-BT accelerometers measured acceleration in three independent dimensions: *X*, *Y*, and *Z*. As the orientation of the devices was consistent among the dogs, these dimensions were oriented along the lateral (*X*), cranio-caudal (*Y*), and dorso-ventral (*Z*) axes of the dogs, respectively. The triaxial acceleration data were sampled at 30 Hz (raw acceleration data), with a dynamic range of ±8.0 g; these parameters were fixed in the commercial availably device. At the end of the study, the raw acceleration data were downloaded from the devices using ActiLife 6^®^ software (ActiGraph, Pensacola, FL, USA) and exported as ‘csv’ files.

### 2.4. Collection and Assessment of the Video Footage

The observation paddock was under constant video surveillance during the day using a 4K security camera system (Swann^®^ Communications USA, Santa Fe Springs, CA, USA). Two cameras were mounted at elevated positions (3.3 m above ground level) on adjacent corners of the observation paddock (Figure 2), allowing for an almost continuously unobstructed view of the dogs throughout the paddock. Video footage was recorded at 15 fps with a resolution of 1920 × 1080, and a bit rate of 2048 kbps.

The behaviour of each of the six dogs was scored continuously (1 s intervals) from the recorded video footage using BORIS^®^ version 7.10.2 [47]. A total of 18 behaviours were scored by a single observer using the ethogram presented in Table 2. These behaviours were categorised as either active (walking, trotting, running, jumping, digging, barking and sniffing), inactive (resting head up, resting head down, sitting, standing, and lateral recumbency) or maintenance (defecating, urinating, eating, drinking and auto grooming), after [25]. In addition, an ‘Other’ category was created to account for behaviours not listed in Table 2 and an ‘Out of sight’ category used for when the dogs could not be seen clearly in the observation paddock.

From the triaxial acceleration data (30 Hz), a total of 32 identifier variables were calculated and summarised into 1 s epochs (Table 3). The correlation between the three accelerometer axes (*XY, XZ, YZ;* three identifier variables), overall dynamic body acceleration (ODBA; one identifier variable), and vector magnitude (VM) were calculated as described in Table 3. For each acceleration axis (*X*, *Y*, and *Z*) and VM, the mean, sum, minimum (min), maximum (max), standard deviation (SD), skewness (skew), and kurtosis (kurt) were calculated.

### 2.5. Building the Behavioural Models

Prior to building the models, the behaviour categories ‘Other’ (*n* = 704) and ‘Out of sight’ (*n* = 1976) were removed from the dataset as they were not representative of a target behaviour. Random forest models were used to develop an algorithm to predict the behaviours of the dogs using the identifier variables calculated from the raw triaxial acceleration data. This method has been successfully used to build predictive behavioural models in other species [21,52,53,54]. In short, RF is a ML technique that builds a large number of decision trees and aggregates them to provide an accurate model for the given classifications [55].

**Figure 3:**
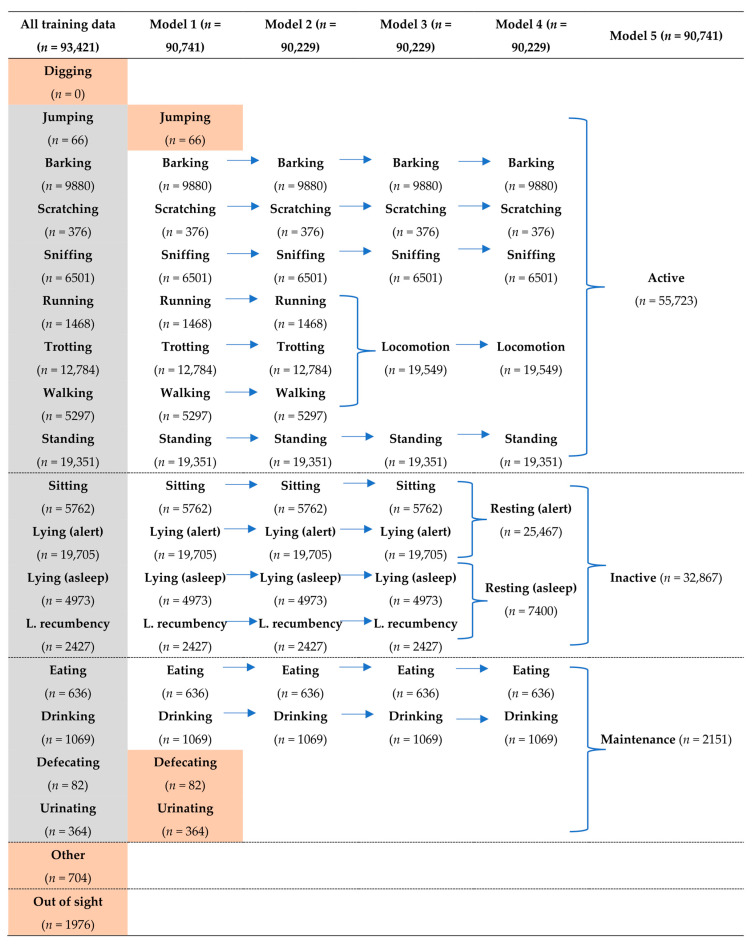
The five modelling rounds showing the total number of observation(s) of each behaviour used for the training data set. The total data set consisted of 129,615 observations, excluding other and out of site categories. Of this data set, 90,741 (70%) and 38,874 observations (30%) were used to train and test the models, respectively. Abbreviations: Lateral (L.) Cells highlighted in orange have been removed from the subsequent models due to low accuracy/precision and/or sample size.

The RF models were built using the packages ‘caret’ and ‘randomForest’ [21] using the default settings of 500 decision trees. Using this method, a total of five models were built from a subset of 70% of the complete dataset with varying levels of behavioural complexity (i.e., number of behaviours assessed) (Figure 3). The behaviours included in each model were selected to determine the ideal balance between model performance and number of behaviours assessed (i.e., level of detail), with a specific emphasis on behaviours that were of biological or clinical significance (e.g., locomotion, scratching, eating, and drinking).

The performance of each model was assessed using the validation dataset, which was the remaining 30% of the complete data set. For each model, Bayesian optimisation of the RF hyperparameters (number of trees, minimum node depth, number of identifier variables randomly sample per tree, and sample fraction) was then conducted using the ‘ranger’ package in R. The RF models were then rebuilt to determine if hyperparameter optimisation could improve the model performance. The relative importance of each identifier variable was determined for each model using the package ‘caret’.

A combination of Synthetic Minority Oversampling Technique (SMOTE) and/or under-sampling [56] was also applied to the data set using the ‘caret’ package in R. All models were then rerun to determine whether model performance could be improved.

### 2.6. Model Evaluation

The performance of each of the five models were compared by constructing and comparing confusion matrices for each model (Supplementary Material Tables S1–S3). From these confusion matrices, the number of observations(s) that were classified as true positive (TP, correctly identified by the model), true negative (TN, not observed and not identified by the model), false positive (FP, identified by the model but not observed) and false negative (FN, observed but not identified by the model) was determined for each behaviour. These data were then used to calculate the sensitivity/recall (the ability of the model to identify TP values), specificity (the ability of the model to identify TN), balanced accuracy positive predictive value or precision (accuracy of positive predictions), precision-recall/F1 score (an accuracy test that is particularly useful for imbalanced data sets; weighted mean of precision and recall), observed prevalence (actual rate of positive observations in the data set), detected prevalence (proportion of observations predicted to be positive) for all behaviours within each of the models (Table 4).

In general, values of >0.7 for parameters such as recall, precision, balanced accuracy, and precision-recall were indicative of sufficient model performance, although the higher these values the better the model performance [21]. The observed prevalence (actual rate of positive observations in the data set) and detected prevalence (proportion of observations predicted to be positive) for all behaviours (Table 4), and the average coefficient of variance (CV%) between the observed prevalence and detected prevalence of each behaviour was then calculated. The overall accuracy of each model was determined by calculating the overall accuracy and the Kappa coefficient (κ; Table 4).

The ActiGraph^®^ accelerometers were validated for the quantification of overall physical activity by comparing the time spent active per hour (as determined by Model 4) with the corresponding sum of ODBA for that hour. These data were compared using a polynomial regression (2nd order). For each behaviour in Model 4 (barking, drinking, eating, locomotion, resting-alert, resting-asleep, scratching, sniffing, and standing), the average ODBA per s was determined. This was then compared, using CV%, against the overage ODBA per s for the same behavioural categories based on the observed behaviour.

## 3. Results

During the study period, all dogs maintained their body weight and no adverse reactions were observed when wearing the devices. From the three days (24 h) of video footage collected from the six dogs, a total of 132,295 s (~36.7 h; ~6.1 h per dog) were scored for observed behaviour. Of the 18 behaviours included in the ethogram (Table 2), ‘digging’ was not observed in the video recordings and was therefore removed from the model building process (Figure 3). Additionally, periods over which the dog’s behaviour was visually classified as ‘other’ (*n* = 704 s) and ‘out of sight’ (*n* = 1,976 s) were also removed. Thus, a total of 90,741 and 38,874 s of scored behavioural data were used as the model training and testing sets, respectively. It has been previously shown that behaviours with large numbers of observations (e.g., >20,000 s) can result in overfitting of the models to these behaviours, thus reducing the overall performance of the model (Smit et al., 2023). In the present study, however, model performance behaviours decreased when behaviours with large numbers of data points were randomly subsampled and limited to 7,000 s (data not presented). As such, all models were built using the complete training data set. The total amount of data used, and the number of behavioural categories, differed depending on the model: Model 1 (16 behavioural categories; 90,741 s), Model 2 (13 behavioural categories; 90,229 s), Model 3 (12 behavioural categories; 90,229 s), Model 4 (nine behavioural categories; 90,229 s), and Model 5 (three behavioural categories; 90,741 s; Figure 3).

### 3.1. Model Performance

A total of five models were built using both the default hyperparameters and Bayes-ian optimised hyperparameters. For all models, there were few differences in the performance of the models when the default or optimised hyperparameters were used. Thus, the default hyperparameters were selected for model building. Furthermore, the overall accuracies and kappa coefficients of the models decreased (by approximately 0.1–0.2) following the application of the SMOTE and random under-sampling relative to the models built using the imbalanced dataset. The use of SMOTE without under-sampling also failed to improve the model performance; thus, the final models presented below were built using the default hyperparameters of the ‘randomForests’ package and the unmodified/imbalanced dataset.

#### 3.1.1. Model 1 (16 Behavioural Categories)

Model 1 included a total of 16 behavioural categories (Table 5) and exhibited an overall accuracy of 0.69 and a κ coefficient of 0.64. While the average specificity was high (0.97 ± 0.01, range 0.86–1.00), the average sensitivity of this model was low compared to the other models (0.60 ± 0.07, range 0.12–0.94). Model 1 had the greatest sensitivity and precision-recall for inactive behaviours (lateral recumbency, lying-alert, and lying-asleep) and sniffing (Table 5). Sensitivity, balanced accuracy, and/or precision-recall values, however, were lower for jumping, running, sitting, standing, trotting, urinating, and walking (Table 5).

From the confusion matrix (Supplementary Material Table S1), it was evident that the model often misclassified behaviours as standing, with standing being the main source of error for eight of the 15 other behaviours assessed. Indeed, the model misclassified 10.5% of drinking, 29.6% of jumping, 54.8% of running, 12.5% of scratching, 24.9% of sitting, 28.5% of trotting, 18.1% of urinating, and 33.6% of walking as standing. The model also misclassified jumping behaviour as barking (14.8% of observations) or trotting (31.2% of observations). Running was also miscategorised as barking by Model 1 (17.4% of observations). In addition sitting behaviour was frequently misclassified as lying-alert (32% of observations). Standing and trotting behaviours were often confused. Urinating was misclassified as both sniffing (21.9% of observations) and standing (18.1% of observations). Lastly, walking behaviour was incorrectly categorised as sniffing (11.9% of observations), standing (33.6% of observations), and trotting (21.6% of observations). While there were other misclassifications of behaviour, these were <10% of observations and will not be discussed here (for more information see Supplementary Material Table S1).

In general, the model struggled to accurately categorise behaviours that were less frequently expressed by the dogs (i.e., prevalence <0.5) (Table 5). The average coefficient of variance between the observed prevalence and detected prevalence of each of the behaviours was 31.4 ± 9.4% (3.0–141.4%). The following behaviours had >20% variance between the observed and detected prevalence: defecating, jumping, running, scratching, sitting, urinating, and walking (Table 5).

#### 3.1.2. Model 2 (13 Behavioural Categories)

For Model 2, three behaviours that were exhibited infrequently and/or had a poor sensitivity in Model 1 were removed: defecating, jumping, and urinating. Thus, Model 2 assessed a total of 13 behavioural categories. In general, the performance of Model 2 was similar to Model 1, with an overall accuracy of 0.69 and a κ coefficient of 0.64. The average sensitivity and specificity for Model 2 were 0.66 ± 0.07 (range, 0.24–0.94) and 0.97 ± 0.01 (range, 0.86–1.00), respectively (Table 5). This led to a slightly higher average balanced accuracy (0.81 ± 0.04) for Model 2 than Model 1. Surprisingly, the average precision-recall of Model 2 (0.69 ± 0.07) was similar to that of Model 1 (0.69 ± 0.05). Model 2 had a precision-recall of <0.70 for running behaviour (0.20), sitting (0.42), standing (0.59), trotting (0.57), and walking (0.34).

The confusion matrix for Model 2 (Supplementary Material Table S2) showed that standing behaviour was again the leading cause for the misclassification of behaviour. Model 2 misclassified 55.1%, 13.8%, 23.9%, 27.9%, and 33.2% of the observed running, scratching, sitting, trotting, and walking behaviour as standing, respectively. Sitting behaviour was also misclassified as lying-alert; 35.7% of observations), and both drinking and standing were frequently misclassified as trotting by the model (12.7% and 19.0% of observations, respectively). In addition, running was often miscategorised as barking (15.0% of observations) and sitting was miscategorised as lying-alert (35.7% of observations; Supplementary Material Table S2). Despite this, Model 2 had a higher balanced accuracy for the less frequent behaviours than Model 1 (Table 5). The average variance between the observed prevalence and detected prevalence of the behaviours was lower than Model 1, with a mean CV% of 21.6 ± 7.3% (range, 3.1–91.7%) for Model 2. The difference between the observed and detected prevalence was greatest for running, sitting, and walking behaviour (Table 5).

#### 3.1.3. Model 3 (11 Behavioural Categories)

As locomotive behaviours (walking, trotting, running) were one of the leading sources of misclassification in Model 2, these behaviours were combined and categorised as locomotion for Model 3. Model 3 assessed a total of 11 behavioural categories and had an overall accuracy of 0.72 and a κ coefficient of 0.66, which was better than Models 1 and 2. The average sensitivity was 0.74 ± 0.05 (range, 0.35–0.94) and the average specificity was 0.96 ± 0.01 (range, 0.89–1.00). While the average specificity for Model 3 was similar to that of Models 1 and 2, the average sensitivity was higher (Table 6). Thus, the average balanced accuracy of Model 3 was also higher (0.85 ± 0.05, range 0.67–0.97) than both Model 1 and Model 2 (Table 6). Sitting was the only behaviour for which the model had poor sensitivity (0.35) and a balanced accuracy less than 0.7 (Table 6). The precision-recall of Model 3, however, was less than 0.7 for the locomotion (0.64), sitting (0.44), and standing (0.58) categories.

It was evident from the confusion matrix (Supplementary Material Table S3) that Model 3 tended to miscategorise sitting behaviour as lying-alert or standing (33.5% and 23.6% of observations, respectively). Interestingly, Model 3 incorrectly classified behaviours as standing less frequently than Models 1 and 2, although observations of locomotion and sitting behaviours were sometimes recorded as standing by the model (21.0% and 23.6% of observations, respectively). The model also miscategorised observations of standing behaviour as locomotion (31.1% of observations). Despite this, the observed and detected prevalences of this model for each of the behaviours agreed, with an average CV% of 11.9 ± 3.5% (range, 0.5–37.2%).

#### 3.1.4. Model 4 (Nine Behavioural Categories)

Given that Model 3 had the lowest accuracy for sitting behaviour and this behaviour was often misclassified as lying-alert, these behaviours were combined and categorised as “resting-alert” for Model 4. In addition, lateral recumbency and lying-resting were combined to form the category “resting-asleep”. Model 4 therefore considered a total of nine behavioural categories. The overall accuracy of Model 4 was 0.74 and the κ coefficient was 0.68; these were higher than all previous models. The average sensitivity for Model 4 (0.76 ± 0.04, range 0.54–0.93) was similar to Model 1, but higher than Models 2 and 3 (Table 5). The specificity for Model 4 (0.96 ± 0.02, range 0.88–1.00) was similar to Models 1, 2, and 3. This ultimately meant that Model 4 had a balanced accuracy and precision-recall that was comparable to Model 3, but higher than Models 1 and 2 (Table 5). In terms of the variation between the observed and detected prevalences, Model 4 had a lower average CV% (10.2 ± 3.0%, range 0–26.6%) than all previous models, with only scratching behaviour having greater than 20% variation between the observed and detected prevalences.

**Figure 4:**
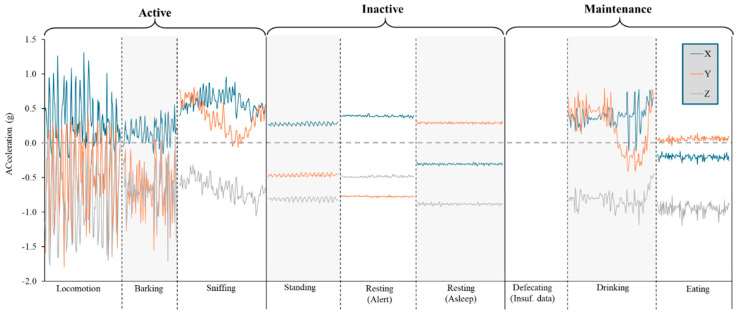
Raw (30 Hz) triaxial (x axis = blue line, y axis = orange line, z axis = grey line) acceleration profiles for each of the behaviours classified by Model 4. A total of 3 s to 5 s present per behaviour, although insufficient continuous acceleration data (Insuf. data) were available for defecation behaviour. These behaviours have been grouped according to the categories used for Model 5: Active, inactive, maintenance.

The confusion matrix for Model 4 (Table 7) showed that the standing and locomotion behaviour were often misclassified, with the observed standing behaviour being classified as locomotion (31.1% of observations) and vice versa (20.7% of observations). Barking, drinking, and scratching behaviour were also occasionally misclassified as locomotion (9.5%, 16.9%, and 11.9% of observations, respectively; Table 7). Eating behaviour was also recorded as sniffing 12.5% of the time by the model (Table 7). Overall, this model accurately distinguished between the assessed behaviours, which was reflected by the low CV% and the raw triaxial acceleration data for each behaviour identified using this model (Figure 4).

#### 3.1.5. Model 5 (Three Behavioural Categories)

The final model (Model 5) was a simplified model consisting of three behavioural categories: active, inactive, and maintenance. This model had by far the highest overall accuracy (0.92) and κ coefficient (0.82) of all tested models. While the average sensitivity (0.84 ± 0.07, range 0.71–0.95) was the highest of all of the models, the average specificity (0.93 ± 0.04, range 0.86–1.0) was the lowest. As a result, the balanced accuracies of Models 5, 4, and 3 were similar, but better than Models 1 and 2 (Table 4). Interestingly, the average precision and precision-recall were much higher for Model 5 (0.98 ± 0.02 and 0.88 ± 0.03, respectively) than all other models (Figure 3). Despite this, the average CV% between the observed and detected prevalences (9.7 ± 6.4%, 2.9–22.4) was similar to Model 4 (Table 6). This was probably because Model 5 often miscategorised inactive and maintenance behaviours as active (13.4% and 26.3% of observations, respectively). Model 5, however, was able to accurately distinguish between the maintenance and inactive categories (Table 8).

### 3.2. Overall Physical Activity/Overall Dynamic Body Acceleration

**Figure 5:**
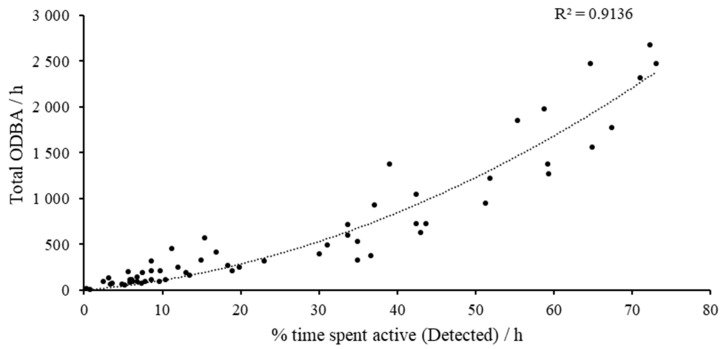
Graph showing the correlation between the time spent active (%) per hour (h) and the total ODBA per hour (h).

Hourly ODBA and the amount of time spent exhibiting active behaviours (barking, locomotion, scratching, sniffing, and standing), as determined by Model 4, were strongly correlated (R^2^ = 0.91, *p* < 0.001: Figure 5). Overall, total ODBA was a significant predictor of the amount of time spent active per hour (*p* < 0.001), and thus, overall physical activity. Indeed, the active behaviours such as barking, and locomotion were associated with the highest average ODBA per second when based on both the observed behavioural data and the detected outputs of Model 4 (Table 9). It was interesting to note that the maintenance behaviours drinking and eating had higher overall ODBA counts per second than sniffing and standing.

## 4. Discussion

This study aimed to validate the use of ActiGraph^®^ accelerometers as a tool for remotely classifying behaviour in domestic dogs. A total of five RF models were built and their performance characteristics compared. Model 4 had an overall accuracy of 74% and was determined to be the optimal model, based on the performance characteristics, confusion matrix, and comparatively low variance between the observed and detected prevalences. This model evaluated a total of nine behavioural categories, namely barking, defecating, drinking, eating, locomotion, resting-asleep, resting-alert, sniffing, and standing. Models such as those developed in the current study are abstract representations of a system, producing approximations of its behaviour [57], which are unlikely to achieve very high accuracy values due to small variations between individuals in their movement and posture. Bardini and others suggest that verification and validation processes should aim at an arbitrarily defined “satisfactory level” of accuracy rather than a maximum [57]; however, there is currently no evidence or consensus on what that satisfactory level should be.

ActiGraph^®^ devices have previously been validated to assess the overall physical activity of animals [15,25,31,58,59,60]. The present study also validated the use of the ActiGraph^®^ for monitoring the overall physical activity, in the form of ODBA, of the dogs. While the use of accelerometery data to assess the expression of specific behaviours has yielded more variable results, the availability of ML techniques has greatly enhanced the potential of building behavioural models for acceleration data [29,60]. Previous studies have reported that using random forests models had 1.5 to 5% higher accuracy in identifying human and animal behaviours than artificial neural network, k-nearest neighbours, linear discriminant analysis, naïve Bayes and support vector machine [33,34]. Behavioural data is often challenging to model as it is inherently imbalanced, with some behaviours (e.g., resting and standing) being expressed far more frequently by the dogs than others (e.g., eating or drinking). When building RF models, it is common to apply SMOTE and/or under-sampling to balance data sets that are originally imbalanced (see review [56]). While in many cases this can improve the model performance, it involves either the removal of data from oversampled categories or generates synthetic data for under-sampled categories [56]. In the present study, it was found that the application of SMOTE to up-sample less frequent behaviours (e.g., drinking, eating, and scratching) and random under-sampling to reduce oversampled behaviours (e.g., locomotion, resting, and standing) reduced model performance when compared to models built using the imbalanced data set. It has previously been shown that under-sampling techniques can reduce model performance when the ratio of imbalance is high [61], as it is for behavioural data. This is largely because the under-sampling process eliminates potentially valuable data and, in turn, increases the variance of the data from the majority classifiers [62]. Dal Pozzolo et al. [62] stated that the efficacy of under-sampling is dependent on both the variance of the classifier and the degree of imbalance between classifiers. Thus, for behavioural data with a high degree of both variance and imbalance, under-sampling may not be appropriate.

The use of SMOTE without under-sampling also failed to improve the model performance in the current study. This was probably because this method generated large amounts of synthetic data produced for behavioural categories with relatively few observations. For less frequent behaviours such a scratching, eating, and drinking, this would mean an up-sampling rate of approximately 18.6, 11.0, and 6.5 times, respectively. Major challenges when applying SMOTE to ML models are overlapping classifiers (in this case behaviours with similar acceleration profiles), a lack of data (e.g., our minority classes), and noise [56]. These challenges are also present in the acceleration data in the present study, which may also contribute to the explanation of why the application of SMOTE failed to improve the model performance. While the use of a balanced data set is optimal for building RF, typical methods for balancing data sets do not seem appropriate for behavioural data and further research into alternative approaches is needed. In the meantime, an emphasis should be placed on performance parameters that are more suited to imbalanced data, such as the F1 or precision-recall scores [63].

Removing behaviours with low sample sizes, provided that they are not of biological or clinical significance, during the model building process may be one approach to dealing with highly imbalanced behavioural data. An alternative option is to group similar behaviours into a single category to increase the sample size for that category. It is known that modelling a wide range of behaviours is challenging, especially if they have a similar acceleration profile [64]. In this context, increasing the number of behavioural classes included in the model has generally been found by us and others to correspond to a decreasing overall accuracy [21,65].

In the present study, there was a progressive increase in the overall accuracies and κ coefficients going from Models 1 to 4 (i.e., from 17 to nine behavioural categories), while the variability between the observed and detected prevalences progressively decreased. Consolidating similar behaviours during model development has also improved the overall accuracy for domestic cat behaviour [21]. For our study, the merging of various locomotor gaits (walking, trotting, and running) into a single locomotion category improved the performance of Model 3 when compared to Model 2. Similarly, consolidating the observed resting behaviours (lateral recumbency, resting-asleep, sitting, and resting-alert) as either resting-alert or resting-asleep further improved the model performance from Model 3 to Model 4. den Uijl et al. [65] also reported that model sensitivity and specificity increased when resting behaviours were broadly categorised as either ‘sleep’ or ‘static’. While combining locomotor gaits and resting behaviours into broader categories improved the model performance, specific target behaviours such as grooming provide valuable health information.

Standing was a consistently difficult behaviour to accurately model, with the misclassification of observations into standing behaviour being one of the leading causes for error in Models 1 to 4 (Supplementary Material Tables S1–S3; Figure 3). Standing had the lowest sensitivity and balanced accuracy of the behaviour categories classified by the optimal model (Model 4), followed by locomotion. Differentiating between the different locomotion gaits and standing was a problem for Models 1 and 2. This was most likely due to the similarities in the position of the device with respect to gravity for active behaviours [66]. In addition, standing is often an intermittent behaviour interspersed with locomotion. Thus, these behaviours are frequently seen within close chronological proximity [49]. Panting, which often occurs with standing behaviour, may also cause movement in the dog’s body, especially in the head area, resulting in unwanted motion [39].

In Model 1, many observed non-locomotor behaviours were also frequently misclassified as standing, including barking, defecating, drinking, eating, sniffing, urinating. While this is not ideal from a model building perspective, it is logical as these behaviours are exhibiting while the dog is standing [49]. This perhaps illustrates the main limitation of the RF approach for modelling behaviour, that is, all modelled behaviours are considered to be independent and mutually exclusive [64]. From a modelling perspective, this means that the RF model can only assign a single behavioural classification for a given timepoint [64]. In reality, many of the behaviours that dogs exhibit, occur simultaneously (e.g., barking and walking, barking and standing, drinking and standing, or eating and standing), which would adversely affect the performance of the model for these behaviours. Thus, we are confronted with a trade-off: either we create an excessive number of behaviour categories, which lowers the model’s accuracy and misclassifies closely related behaviours, or we have too few categories to adequately represent the full range of the species’ behaviours.

In the initial models (Models 1 to 3), many of the static or resting behaviours (e.g., standing, lying-alert, and lying-asleep) were also misclassified, despite being consider as mutually exclusive. For example, sitting was often misclassified as other static behaviours such as lying-alert and standing. Kumpulainen et al. [29] also noted there were challenges in differentiating static postures such as lying down, sitting, and standing from triaxially acceleration data. It has been hypothesised that the minimal changes in neck and back orientation during these behaviours might be too subtle for the device to distinguish [29,65]. Alternatively, unwanted rotation of the device and collar around the neck could also be problematic for distinguishing between static behaviours.

Our previous study in cats showed that RF and self-organising map (SOM) models built for collar-mounted devices generally performed worse than those fitted to a harness, although both the final collar and harness models were considered satisfactory [21]. Many authors have attributed lower model performance to an increase in the residual movement of the devices (i.e., continued movement after a behaviour has stopped) and/or the rotation of the devices around the collar, leading to changes in device orientation [21,29,67,68].

Westgarth and Ladha [68] and one of our previous studies [21] stated that harness-attached accelerometers might be advantageous due to their inability to rotate. Not all dogs, however, are accustomed to, or willing, to wear harnesses over a long period [68]. Given this and the practical simplicity of collar attachment, many pet owners would likely prefer a collar-attached device. Indeed, in our previous study of cat owners chose either a harness or collar attachment of ActiGraph^®^ devices, with the majority selecting the collar attachment [69]. Whether this is the case for dogs remains to be investigated.

Regardless of the attachment method, it is clear from our previous study that RF can accurately assess the behaviour of animals from continuous triaxial acceleration data [21], but caution is needed when constructing the models to determine an appropriate balance between detail (number of behaviours) and performance. Thus, our approach of progressively simplifying the RF model over several modelling rounds, as in the present study and that of Smit et al. [21], is an important aspect of building behavioural algorithms for acceleration data. While many behaviours were misclassified in the initial models of the present study (especially Model 1), the optimal model (Model 4) showed an acceptable level of accuracy and precision.

The performance characteristics obtained from the confusion matrices provide useful insight during the model-building process. From a practical perspective, however, the key feature of a good model is that it accurately predicts the percentage of time an animal spent exhibiting each behaviour over a given time point. Indeed, our previous studies utilising these models to assess factors affecting animal behaviour have concentrated on the percentage of time spent exhibiting each behaviour per hour, day, or week [69,70]. The final model in the current study (Model 4) showed a high amount of agreement between the observed prevalence (proportion time spent exhibiting each behaviour based on actual observations) and detection prevalence (the proportion of time spend exhibiting each behaviour based on the model classifications) of most behaviours. Thus, this model provided an accurate and reliable assessment of canine behaviour from the triaxial acceleration data.

Future studies should investigate whether the addition of technologies such as gyroscopes alongside a triaxial accelerometer could enhance the performance of RF models for canine behaviour. The effect of sampling frequency and dynamic range of the triaxial acceleration data should also be investigated, although these parameters were fixed in the present study. Those looking to develop novel accelerometer-based units for monitoring animal behaviour should consider these factors thoroughly. It would also be worth investigating whether time series ML approaches such as long short-term memory (LSTM) would result in improved model performance.

## 5. Conclusions

This study successfully validated the use of the ActiGraph^®^ wGT3X-BT accelerometers as a tool for remotely classifying behaviours in domestic dogs. Using ML to build five RF models, Model 4 was identified as the optimal model for dog behaviour research. This model encompassed nine behavioural classification categories (barking, defecating, drinking, eating, locomotion, resting-asleep, resting-alert, sniffing, and standing), demonstrated an overall accuracy of 74% whilst maintaining a behavioural repertoire that would be useful for a range of different study objectives.

The results support previous validations of ActiGraph^®^ devices for measuring overall physical activity in animals. This study also expanded the validation to specific behaviours by using machine learning techniques to improve the model’s accuracy. Despite the difficulty of modelling a variety of behaviours with similar acceleration patterns, grouping similar behaviours together was an effective approach.

The use of ActiGraph^®^ accelerometers combined with refined RF models offers a promising method for detailed and remote assessment of canine behaviour. Although challenges remain in accurately classifying similar behaviours, advancement holds the potential for improving our understanding of animal behaviour and enhancing the welfare of domestic dogs through better monitoring and analysis.

## Figures and Tables

**Table 1:** Description of study dogs (*n* = 6) including name, sex, age, breed and reproductive status.

Name	Sex	Pair	Age (years)	Breed	Desexed	Weight (kg)
Belvedere	Female	3	7.5	Huntaway	Yes	22.4
Blacky	Male	3	3.9	Huntaway/Heading	Yes	23.9
Chevelle	Female	2	7.5	Huntaway	Yes	23.0
Gizmo	Male	1	5.7	Harrier Hound	Yes	31.1
Gus	Male	2	4.0	Huntaway/Smithfield Terrier	Yes	22.7
Monaro	Male	1	7.5	Huntaway	Yes	32.8

**Table 2:** Ethogram of defined canine behaviours and their categorisation as either active, inactive, or maintenance.

Category	Behaviour	Description
Active	Walking	The slowest upright gait where the body is moving forward, each paw lifting from the ground one at a time in a regular sequence [48].
	Trotting	A rhythmic two-beat gait where diagonally opposite paws strike the ground at the same time as the subject moves forward. This gait is faster than walking [48].
	Running	Can also be defined as a ‘canter’. This is a three-beat gait in which two legs move separately and two as a diagonal pair. This gait is faster than a walk and trot [48].
	Jumping	Subject has both hindlegs on the floor and rears in a manner that results in both forelegs in contact with the fencing of paddock, kennel, or person [49].
	Barking	Barking is defined as the mouth being opened and closed quickly in a snapping motion, releasing a low frequency vocalization [49].
	Sniffing	Nose directed to a point of interest and sniffs [50].
	Digging	The dog uses its forepaws to repeatedly scratch the ground surface [49].
	Scratching	Grooming behaviour directed towards subjects’ own body, using paws [49].
Inactive	Resting-alert	Lying on stomach with forelegs extended to the front, hind legs bent and resting close to the body on each side, or with the body twisted and both hind legs on one side. Head is held up off the ground or surface [47].
	Resting-asleep	Lying on stomach with forelegs extended to the front, hind legs bent and resting close to the body on each side, or with the body twisted and both hind legs on one side. Head is lowered to rest on either forelegs or the ground between them [47].
	L. recumbency	Lying down flat on one side with head resting on surface in sideways position [51].
	Sitting	Hind quarters on ground with front legs standing up straight and being used for support [49].
	Standing	All four paws planted on ground and legs extended so they are upright in stationary position [49].
Maintenance	Defecating	Excretion of faeces from the subject’s body [49].
	Urinating	Excretion of urine from the subject’s body [49].
	Eating	Subject chews and ingests food from bowl provided by human [49].
	Drinking	Subject drinks from water bowl in paddock by lapping up the water with their tongue [48,49].
	Auto grooming	Grooming behaviour directed towards the subject’s own body including licking, self-biting, and scratching [49].
Other	Other	Any behaviour that does not fit into one of the behaviours included in this ethogram.
	Out of sight	Subject is out of view and behaviour cannot be observed.

Abbreviation: Lateral recumbency (L. Recumbency).

**Table 3:** Description of identifier variables for model building.

Identifier Variable	Description
Mean acceleration	Mean which is calculated for every second using the raw acceleration data (30 measures per second)
Sum acceleration	Sum_(Axis)_ = ∑ Axisi
Minimum (min)	Minimum value of every 30 measures per second
Maximum (max)	Maximum value of every 30 measures per second
Standard deviation (SD)	Quantifies the amount of variability within a dataset
Skewness	Measures the asymmetry of the probability distribution of a dataset
Kurtosis	Measures the weight of the tails in relation to normal distribution
Vector magnitude (VM)	V M = √ *X*^2^ + *Y*^2^ + *Z*^2^
Overall dynamic body acceleration (ODBA)	*ODBA* = $\sum_{i=1}^{n}\left|{DBA}_{X}|+|{DBA}_{Y}|+|{DBA}_{Z}\right|$
Dynamic body acceleration (DBA)	DBA = Sum_axis_ − moving average

**Table 4:** Calculations for the parameters used to assess the performance of the identifier variables.

Parameter	Calculation
Sensitivity/recall	=TP/(TP + FN)
Specificity	=TP/(TN + FP)
Balanced accuracy	=(*sensitivity + specificity*)/2
Precision	=TP/(TP + FP)
Precision recall (F1 Score)	=2×((Precision×Sensitivity)/(Precision+Sensitivity))
Observed prevalence	=(TP + FN)/(TP + TN + FP + FN)
Detected prevalence	=(TP + FP)/(TP + TN + FP + FN)
Overall accuracy	=(TP + TN)/(TP + TN + FP + FN)
Kappa coefficient (κ)	$=(N\;x\;\sum_{i=1}^{k}x_{ii}-\sum_{i=1}^{k}x_{i}(x_{i+}\times \;x_{+i}))/N^{2}-\sum_{i=1}^{k}(x_{i+}\times \;x_{+i})\;$ where *N* = Total number of observations (all behaviours) *k* = Number of behaviour categories *i* = Behaviour category *i* *x_ii_ =* Number of observations that both that both visual observation and the predictor model classified into the *i*-th category *x_i+_ =* Number of observations that were visually classified into the *i*-th category
	*X_+i_* = Number of observations that the predictor model classified into the *i*-th category

Abbreviations: true positive (TP), true negative (TN), false positive (FP), false negative (FN).

**Table 5:** The performance characteristics of the random forest Models one and two showing the sensitivity (proportion of true positives), specificity (proportion of true negatives), balanced accuracy (average proportion of sensitivity and specificity), prevalence (proportion of time the animals were observed (visually) or detected (by the model) exhibiting a given behaviour) and the coefficient of variance (CV%) between the observed and detected prevalence (CV% = SD/mean × 100). Behaviours highlighted in red have a CV% > 20. Cells highlighted in orange reflect performance characteristics that scored <0.70.

Behaviour	Sensitivity	Specificity	Balanced Accuracy	Precision	Precision-Recall	Prevalence (Observed)	Detection Prevalence	CV%
Model 1								
Barking	0.84	0.97	0.91	0.80	0.82	0.109	0.115	3.77
Defecating	0.71	1.00	0.85	0.96	0.81	0.001	0.001	21.57
Drinking	0.71	1.00	0.85	0.92	0.80	0.012	0.009	18.75
Eating	0.78	1.00	0.89	0.96	0.86	0.007	0.006	14.95
Jumping	0.00	1.00	0.50	-	-	0.001	0.000	141.42
L. recumbency	0.94	1.00	0.97	0.98	0.96	0.027	0.026	2.83
Lying-asleep	0.85	1.00	0.92	0.91	0.88	0.055	0.051	4.67
Lying-alert	0.85	0.94	0.89	0.79	0.82	0.217	0.234	5.11
Running	0.12	1.00	0.56	0.54	0.20	0.016	0.004	88.67
Scratching	0.61	1.00	0.80	0.97	0.75	0.004	0.003	32.65
Sitting	0.37	0.99	0.68	0.63	0.47	0.064	0.037	36.54
Sniffing	0.93	0.98	0.95	0.75	0.83	0.072	0.089	15.06
Standing	0.64	0.86	0.75	0.55	0.59	0.213	0.251	11.57
Trotting	0.58	0.92	0.75	0.56	0.57	0.141	0.147	3.04
Urinating	0.48	1.00	0.74	1.00	0.65	0.004	0.002	49.20
Walking	0.25	0.99	0.62	0.54	0.34	0.058	0.027	52.88
Average	0.60 ± 0.07	0.97 ± 0.01	0.79 ± 0.04	0.34 ± 0.08	0.79 ± 0.05	⅀ = 1.000	⅀ = 1.000	31.4 ± 9.4
Model 2								
Barking	0.84	0.97	0.90	0.80	0.82	0.110	0.115	3.25
Drinking	0.71	1.00	0.86	0.91	0.80	0.012	0.009	17.18
Eating	0.79	1.00	0.90	0.93	0.86	0.007	0.006	11.23
L. Recumbency	0.94	1.00	0.97	0.98	0.96	0.027	0.026	3.12
Lying-asleep	0.85	1.00	0.92	0.92	0.88	0.055	0.051	5.27
Lying-alert	0.85	0.93	0.89	0.78	0.81	0.218	0.238	6.07
Running	0.12	1.00	0.56	0.56	0.20	0.016	0.003	91.69
Scratching	0.72	1.00	0.86	0.95	0.82	0.004	0.003	19.63
Sitting	0.33	0.98	0.66	0.58	0.42	0.064	0.037	37.87
Sniffing	0.94	0.98	0.96	0.77	0.85	0.072	0.087	13.42
Standing	0.64	0.86	0.75	0.55	0.59	0.215	0.248	10.13
Trotting	0.59	0.92	0.76	0.55	0.57	0.142	0.152	5.02
Walking	0.24	0.99	0.61	0.56	0.34	0.059	0.025	57.21
Average	0.66 ± 0.07	0.97 ± 0.01	0.81 ± 0.04	0.76 ± 0.05	0.69 ± 0.07	⅀ = 1.000	⅀ = 1.000	21.6 ± 7.3

**Table 6:** The performance characteristics of the random forest Models three, four and five showing the sensitivity (proportion of true positives), specificity (proportion of true negatives), balanced accuracy (average proportion of sensitivity and specificity), prevalence (proportion of time the animals were observed (visually) or detected (by the model) exhibiting a given behaviour) and the coefficient of variance (CV%) between the observed and detected prevalence (CV% = SD/mean × 100). Behaviours highlighted in red have a CV% > 20. Cells highlighted in orange reflect performance characteristics that scored <0.70.

Behaviour	Sensitivity	Specificity	Balanced Accuracy	Precision	Precision-Recall	Prevalence (Observed)	Detection Prevalence	CV%
Model 3								
Barking	0.82	0.98	0.90	0.83	0.83	0.110	0.109	0.49
Drinking	0.71	1.00	0.86	0.93	0.81	0.012	0.009	18.95
Eating	0.79	1.00	0.90	0.97	0.87	0.007	0.006	14.32
L. Recumbency	0.94	1.00	0.97	0.99	0.96	0.027	0.025	4.11
Locomotion	0.67	0.88	0.77	0.61	0.64	0.217	0.236	6.09
Lying-asleep	0.87	1.00	0.93	0.92	0.89	0.055	0.052	4.13
Lying-alert	0.84	0.94	0.89	0.79	0.81	0.218	0.233	4.57
Scratching	0.64	1.00	0.82	0.94	0.77	0.004	0.003	26.83
Sitting	0.35	0.98	0.67	0.60	0.44	0.064	0.037	37.22
Sniffing	0.93	0.98	0.95	0.79	0.85	0.072	0.084	11.20
Standing	0.57	0.89	0.73	0.59	0.58	0.215	0.205	3.06
Average	0.74 ± 0.05	0.96 ± 0.01	0.85 ± 0.03	0.82 ± 0.05	0.77 ± 0.05	⅀ = 1.000	⅀ = 1.000	11.9 ± 3.5
Model 4								
Barking	0.82	0.98	0.90	0.83	0.82	0.110	0.108	0.70
Drinking	0.73	1.00	0.86	0.93	0.82	0.012	0.009	17.37
Eating	0.72	1.00	0.86	0.96	0.82	0.007	0.005	19.87
Locomotion	0.66	0.88	0.77	0.61	0.64	0.217	0.234	5.37
Rest-asleep	0.89	1.00	0.94	0.94	0.92	0.082	0.077	4.11
Rest-alert	0.85	0.94	0.89	0.85	0.85	0.282	0.283	0.08
Scratching	0.66	1.00	0.83	0.96	0.79	0.004	0.003	26.19
Sniffing	0.93	0.98	0.95	0.79	0.85	0.072	0.085	11.51
Standing	0.54	0.90	0.72	0.59	0.57	0.215	0.196	6.52
Average	0.76 ± 0.04	0.96 ± 0.02	0.86 ± 0.03	0.83 ± 0.05	0.78 ± 0.04	⅀ = 1.000	⅀ = 1.000	10.2 ± 3.0
Model 5								
Active	0.95	0.86	0.9053	0.91	0.93	0.613	0.638	2.9
Inactive	0.87	0.95	0.9095	0.91	0.89	0.364	0.345	3.8
Maintenance	0.71	1.00	0.85618	0.98	0.83	0.023	0.017	22.4
Average	0.84 ± 0.07	0.93 ± 0.04	0.86 ± 0.04	0.98 ± 0.02	0.88 ± 0.03	⅀ = 1.000	⅀ = 1.000	9.7 ± 6.4

**Table 7:** Confusion matrix of predicted and observed observations (s) from Model 4 presented as percentages (%). Correct categorisations by the model are indicated in cells highlighted in green and incorrect categorisations >10% are in cells that have been highlighted in orange.

Model Prediction	Observed Behaviour								
	Barking	Drinking	Eating	Locomotion	Resting-Asleep	Resting-Alert	Scratching	Sniffing	Standing
Barking	81.77	0.44	2.94	4.20	0.19	1.24	0.00	0.07	2.71
Drinking	0.00	72.65	0.00	0.18	0.00	0.00	0.00	0.00	0.12
Eating	0.00	0.00	72.06	0.02	0.00	0.01	0.00	0.14	0.02
Locomotion	9.59	16.85	5.88	66.22	0.41	2.59	11.88	3.59	31.08
Resting-asleep	0.09	0.00	0.00	0.20	89.06	1.11	1.88	0.22	0.21
Resting-alert	4.53	0.44	2.21	4.81	8.01	84.89	8.75	0.72	9.29
Scratching	0.00	0.00	0.00	0.01	0.03	0.02	66.25	0.00	0.00
Sniffing	0.09	4.81	12.50	3.69	0.57	0.82	2.50	92.89	2.54
Standing	3.92	4.81	4.41	20.66	1.73	9.33	8.75	2.37	54.03
n	4234	457	272	8377	3171	10,914	160	2785	8292

**Table 8:** Confusion matrix of predicted and observed behaviours for Model 5 presented as percentages (%). Correct categorisations by the model are indicated in cells highlighted in green and incorrect categorisations >10% are in cells that have been highlighted in orange.

Model Predictions	Observed Behaviour		
	Active	Inactive	Maintenance
Active	95.22	13.40	26.26
Inactive	4.75	86.57	2.47
Maintenance	0.03	0.04	71.27
Total Observations (s)	23,690	14,085	891

**Table 9:** Table showing both the observed and detected average ODBA/s as well as the coefficient of variance (CV) for each behaviour. Behaviours were selected from Model 4 (9 behavioural categories).

Behaviour	Average ODBA/s (Observed)	Average ODBA/s (Detected)	CV%
Locomotion	0.819 ± 0.007	0.996 ± 0.006	13.8
Barking	0.726 ± 0.015	0.707 ± 0.014	1.9
Drinking	0.573 ± 0.032	0.659 ± 0.047	9.9
Eating	0.514 ± 0.031	0.427 ± 0.031	13.1
Standing	0.454 ± 0.006	0.277 ± 0.004	34.2
Scratching	0.372 ± 0.057	0.465 ± 0.075	15.7
Sniffing	0.364 ± 0.008	0.357 ± 0.006	1.4
Resting-alert	0.069 ± 0.001	0.047 ± 0.001	26.8
Resting-asleep	0.020 ± 0.001	0.014 ± 0.000	25.3
Average	0.435 ± 0.089	0.439 ± 0.105	15.7 ± 3.7

## Data Availability

A dataset including the weekly behavioural counts and percentages can be made available upon request.

## Supplementary Materials

The following supporting information can be downloaded at: https://www.mdpi.com/article/10.3390/s24185955/s1, Table S1: Confusion matrix of predicted and observed behaviours of Model 1 presented as percentages (%). Correct/target categorisations by the model are indicated in cells highlighted green and incorrect categorisations > 10% are in cells that have been highlighted orange. Abbreviation: Lateral recumbency (Lateral R.); Table S2: Confusion matrix of predicted and observed behaviours of Model 2 presented as percentages (%). Correct/target categorisations by the model are indicated in cells highlighted green and incorrect categorisations >10% are in cells that have been highlighted orange. Abbreviation: Lateral recumbency (L. recumbency); Table S3: Confusion matrix of predicted and observed behaviours of Model 3 presented as percentages (%). Correct/target categorisations by the model are indicated in cells highlighted green and incorrect categorisations >10% are in cells that have been highlighted orange. Abbreviation: Lateral recumbency (Lateral R.).
