# Supplementary material for: The Use of Triaxial Accelerometers and Machine Learning Algorithms for Behavioural Identification in Domestic Dogs (Canis familiaris): A Validation Study
Source: Sensors (Basel). 2024 Sep 13;24(18):5955. doi: 10.3390/s24185955 (PMC11435861; doi:10.3390/s24185955)
Supplement: Supplementary file 1 [file sensors-24-05955-s001.zip › sensors-3101028-SI.pdf]

Table S1: Confusion matrix of predicted and observed behaviours of Model 1 presented as percentages (%). Correct/target categorisations by the model are indicated in cells highlighted green and incorrect categorisations >10% are in cells that have been highlighted orange. Abbreviation: Lateral recumbency (Lateral R.).

Model Prediction	Observed behaviour															
	Barking	Defecating	Drinking	Eating	Jumping	Lateral R.	Lying (asleep)	Lying (alert)	Running	Scratching	Sitting	Sniffing	Standing	Trotting	Urinating	Walking
**Barking**	83.89	0.00	0.00	2.94	14.81	0.10	0.19	0.96	17.36	5.00	2.84	0.00	3.46	5.39	0.00	2.07
**Defecating**	0.00	70.59	0.00	0.00	0.00	0.00	0.00	0.00	0.00	0.00	0.00	0.00	0.01	0.00	0.00	0.00
**Drinking**	0.00	0.00	70.68	0.00	0.00	0.00	0.00	0.00	0.00	0.00	0.00	0.00	0.16	0.07	0.00	0.44
**Eating**	0.00	0.00	0.00	77.94	0.00	0.00	0.05	0.00	0.00	0.00	0.00	0.07	0.04	0.02	0.00	0.04
**Jumping**	0.00	0.00	0.00	0.00	0.00	0.00	0.00	0.00	0.00	0.00	0.00	0.00	0.00	0.00	0.00	0.00
**L. recumbency**	0.00	0.00	0.00	0.00	0.00	94.13	0.19	0.13	0.00	0.00	0.08	0.00	0.02	0.00	0.00	0.04
**Lying-asleep**	0.05	5.88	0.00	0.00	0.00	0.48	85.40	1.34	0.16	0.00	0.16	0.00	0.28	0.13	1.29	0.71
**Lying-alert**	2.88	0.00	0.88	0.00	7.41	3.56	9.67	84.96	5.41	5.63	31.15	0.65	5.40	3.16	0.00	3.53
**Running**	0.28	0.00	0.00	0.00	3.70	0.00	0.00	0.00	12.42	0.00	0.00	0.07	0.41	0.27	1.29	0.00
**Scratching**	0.00	0.00	0.00	0.00	0.00	0.00	0.00	0.02	0.00	60.63	0.00	0.00	0.01	0.00	0.00	0.00
**Sitting**	0.57	0.00	0.44	0.00	3.70	0.38	0.38	2.90	2.71	1.25	37.02	0.43	1.88	0.69	0.00	1.41
**Sniffing**	0.09	5.88	6.35	14.71	3.70	0.58	1.08	0.86	1.59	6.88	1.46	92.85	2.79	1.70	21.94	11.90
**Standing**	5.43	5.88	10.50	4.41	29.63	0.67	2.68	7.79	54.78	12.50	24.91	3.02	64.32	28.48	18.06	33.58
**Trotting**	6.57	5.88	7.88	0.00	33.33	0.10	0.23	0.64	5.25	6.88	1.22	1.08	18.51	58.38	5.16	21.55
**Urinating**	0.00	0.00	0.00	0.00	0.00	0.00	0.00	0.00	0.00	0.00	0.00	0.00	0.00	0.00	48.39	0.00
**Walking**	0.24	5.88	3.28	0.00	3.70	0.00	0.14	0.39	0.32	1.25	1.17	1.83	2.71	1.72	3.87	24.72
**Total observations (s)**	**4,234**	**34**	**457**	**272**	**27**	**1,040**	**2,130**	**8,444**	**628**	**160**	**2,469**	**2,785**	**8,292**	**5,478**	**155**	**2,269**

Table S2: Confusion matrix of predicted and observed behaviours of Model 2 presented as percentages (%). Correct/target categorisations by the model are indicated in cells highlighted green and incorrect categorisations >10% are in cells that have been highlighted orange. Abbreviation: Lateral recumbency (L. recumbency).

Model prediction	Observed behaviour											
	Barking	Drinking	Eating	Lateral R.	Lying (asleep)	Lying (alert)	Running	Scratching	Sitting	Sniffing	Standing	Trotting	Walking
**Barking**	83.56	0.00	2.94	0.29	0.00	0.83	14.97	3.13	2.96	0.00	3.49	5.59	2.07
**Drinking**	0.00	71.12	0.00	0.00	0.00	0.00	0.00	0.00	0.00	0.00	0.18	0.13	0.48
**Eating**	0.00	0.00	79.41	0.00	0.00	0.00	0.00	0.00	0.00	0.29	0.04	0.04	0.13
**L. recumbency**	0.00	0.00	0.00	94.13	0.05	0.08	0.00	0.00	0.16	0.00	0.01	0.00	0.13
**Lying-asleep**	0.00	0.00	0.00	0.38	85.31	1.30	0.16	1.25	0.20	0.07	0.19	0.11	0.62
**Lying-alert**	3.16	0.66	0.00	3.56	10.47	84.81	5.41	6.25	35.68	0.50	5.73	2.76	3.35
**Running**	0.14	0.00	0.00	0.00	0.00	0.00	11.94	0.00	0.00	0.00	0.37	0.33	0.18
**Scratching**	0.00	0.00	0.00	0.00	0.00	0.06	0.00	71.88	0.04	0.00	0.00	0.00	0.00
**Sitting**	0.61	0.88	0.00	0.38	0.14	3.19	2.39	1.25	33.50	0.22	2.34	0.64	1.81
**Sniffing**	0.14	6.13	13.24	0.48	0.75	0.75	1.27	0.00	1.17	93.57	2.76	1.52	11.46
**Standing**	5.05	6.35	3.68	0.67	3.10	7.83	55.10	13.75	23.94	2.62	63.57	27.89	33.19
**Trotting**	7.09	12.69	0.00	0.00	0.19	0.90	8.60	1.88	1.13	1.01	18.96	59.22	22.65
**Walking**	0.24	2.19	0.74	0.10	0.00	0.26	0.16	0.63	1.22	1.72	2.36	1.79	23.93
**Total observations (s)**	**4,234**	**457**	**272**	**1,040**	**2,130**	**8,444**	**628**	**160**	**2,469**	**2,785**	**8,292**	**5,478**	**2,269**

Table S3: Confusion matrix of predicted and observed behaviours of Model 3 presented as percentages (%). Correct/target categorisations by the model are indicated in cells highlighted green and incorrect categorisations >10% are in cells that have been highlighted orange. Abbreviation: Lateral recumbency (Lateral R.).

Model prediction	Observed behaviour									
	Barking	Drinking	Eating	Lateral R.	Locomotion	Lying (asleep)	Lying (alert)	Scratching	Sitting	Sniffing	Standing
**Barking**	82.43	0.44	2.21	0.19	4.03	0.19	0.68	3.13	2.55	0.29	2.77
**Drinking**	0.00	71.33	0.74	0.00	0.13	0.00	0.00	0.00	0.00	0.07	0.10
**Eating**	0.00	0.00	79.41	0.00	0.02	0.00	0.01	0.00	0.00	0.07	0.01
**L. recumbency**	0.00	0.00	0.00	93.56	0.00	0.05	0.07	0.00	0.00	0.00	0.01
**Locomotion**	9.78	16.63	8.09	0.58	66.66	0.56	2.40	14.38	3.93	4.09	31.14
**Lying-asleep**	0.09	0.00	0.00	0.58	0.14	86.76	1.42	1.25	0.12	0.07	0.14
**Lying-alert**	3.71	0.00	0.74	3.85	3.08	8.59	84.13	8.75	33.54	0.57	4.90
**Scratching**	0.00	0.00	0.00	0.00	0.00	0.00	0.06	64.38	0.04	0.00	0.00
**Sitting**	0.43	1.31	0.00	0.58	1.10	0.38	3.33	2.50	34.95	0.29	1.86
**Sniffing**	0.14	3.06	6.62	0.29	3.83	0.75	0.68	2.50	1.26	92.82	2.52
**Standing**	3.42	7.22	2.21	0.38	21.00	2.72	7.22	3.13	23.61	1.72	56.55
**Total Observations(s)**	**4,234**	**457**	**272**	**1,040**	**8,377**	**2,130**	**8,444**	**160**	**2,469**	**2,785**	**8,292**
